# Fabrication of Photo-Responsive Mesh Membrane with Surface-Engineered Wettability for Oil–Water Separation and Photocatalytic Degradation of Organic Pollutants

**DOI:** 10.3390/membranes13030302

**Published:** 2023-03-04

**Authors:** Umair Baig, Mohamed A. Dastageer

**Affiliations:** 1 IRC-Membranes and Water Security, King Fahd University of Petroleum and Minerals, Dhahran 31261, Saudi Arabia; 2 Department of Physics, King Fahd University of Petroleum and Minerals, Dhahran 31261, Saudi Arabia

**Keywords:** mesh membrane, TiO_2_ nanoparticles, surface wettability, oil–water separation, photocatalysis

## Abstract

A photo-responsive TiO_2_-coated stainless-steel mesh membrane (TiO_2_@SSM), possessing unique surface wettability, was fabricated. This TiO_2_@SSM membrane is found to be capable of separating oil and water from oily water and has the potential to carry out photocatalytic self-cleaning and/or the degradation of organic pollutants present in water. The fabrication of TiO_2_@SSM is quite simple: titanium dioxide (TiO_2_) nanoparticles were spray-coated onto stainless steel microporous mesh (SSM) substrates and annealed at the temperature of 500 °C. The fabricated TiO_2_@SSM membrane was structurally and morphologically characterized by XRD, FE-SEM, EDX, and elemental mapping. The contact angle measurements using a goniometer showed that the fabricated TiO_2_@SSM membrane surface is superhydrophilic and superoleophilic in air and superoleophobic under water. This is a favorable wetting condition for the water passing oil–water separation membrane, and this water passing property of the membrane eased the common problem of the fast clogging of the membrane by oil. An oil–water separation efficiency of about 99% was achieved, when the TiO_2_@SSM membrane was used as the separating medium in the gravity-driven oil–water separation system, unlike the uncoated stainless steel mesh membrane, which allowed both oil and water to pass together. This confirmed that the oil–water separating functionality of the membrane is attributed to TiO_2_ coating on the stainless steel mesh. The photocatalytic degradation property of the TiO_2_@SSM membrane is an added advantage, where the membrane can be potentially used for self-cleaning of the membrane’s surface and/or for water purification.

## 1. Introduction

There are many sources for the mixing of oil in water, such as accidental and intentional leakage from oil tankers into the sea, the release of waste oil into water bodies, and the produced water generated during oil excavation [1,2]. The oily water thus generated poses a great deal of menace to the environment in general, and the lives of those in the marine environment in particular [3]. Growing global awareness and initiatives to protect the environment has urged countries to enact stringent legislations to keep this reckless manipulation of the environment in check [4]. The generation of oily water from the petrochemical industries and oil rigs are quite inevitable as a huge amount of water is poured into oil wells, particularly into ageing oil wells in order to obtain a high yield in oil excavation [5]. Oil companies are forced to deal with the oily water in order to protect the fragile environment and also to redeem a huge quantity of reusable water as a spin-off. Many viable technologies, based on physical, chemical, and biological methods such as magnetic separation, chemical separation, centrifuging, filtration, floatation, and separation with oil absorbents, have been developed for separating oil and water from oily water [6,7,8]. In addition to oil contamination, water is polluted with other organic/inorganic substances and microorganisms such as sulfate-reducing bacteria (SRB), which need to be purified after oil–water separation [9,10].

Membrane-based filtering systems have been developed for many purification applications including oil–water separation [6,7,8,11]. Many variants of specially fabricated microporous and nanoporous membranes have proven to be less effective for oil–water separation because of the rapid clogging of pores by oil and oily water. In order to circumvent these problems, membranes with preferential wettability for water and oil were developed [12,13,14,15]. The wettability of a particular liquid is decided by the relative magnitudes of the interfacial surface energy of the membrane surface and the surface tension of the liquid. When surface energy is more than the surface tension of the liquid, the adhesive force of the liquid on the surface is more than the cohesive force of the liquid, which leads to the wetting of the surface, and on the other hand, when the liquid surface tension is dominant, the cohesive force overpowers the adhesive force, which leads to the beading of liquid on the surface. Therefore, in order to achieve a surface with the desired wettability for oil and water, the chemical composition of the coated material and the hierarchical surface roughness are factored in. The membrane surfaces, showing a high affinity for oil and a strong repulsion for water (superhydrophobic/superoleophilic), were fabricated by many groups for the application of oil–water separation [16,17]. The superhydrophobic/superoleophilic membranes permeated oil through the pores due to a strong oil affinity and retained water with a separation efficiency as high as 95%. Nevertheless, the stability and the sustainability of membranes that pass oil remain to be a real technical challenge due to the rapid clogging of pores by oil. The second kind of surface wettability contemplated for oil–water separation was the membrane surface, which showed a high affinity for water and a strong repulsion of oil (superhydrophilic/superoleophobic) [6,7,8]. Although the realization of the membrane surfaces with a second kind of wettability is an apparent solution to minimize oil clogging in the membrane’s pores, a material showing this kind of surface wettability is difficult to achieve due to the fact that the surface tension of water is almost twice that of oil, and also according to the basic laws of wettability.

However, it is possible to develop an in-air superhydrophilic/superoleophilic membrane surface, which drastically flips its oil wettability to superoleophobic under the water by selecting the right material and also by engineering the surface roughness [14,15,18]. In this kind of membrane, a positive upward oil pressure due to solid–water–oil interfacial tension pushed the oil away from the membrane’s surface and a negative downward water pressure due to solid–water–air interfacial tension pushed the water down. As this membrane is water passing, the oil clogging problem is drastically reduced, which renders a high stability and reusability for the membrane. Metal oxides, metal organic frameworks (MOF), metal carbides, and zeolites have been tried in attempt to achieve superhydrophilic–underwater superoleophobic wettability [18]. In addition to this, in some recent works, polymeric materials showed superhydrophilic–underwater superoleophobic properties, and these membranes showed a good oil–water separation efficiency, but polymeric materials are fragile and are not durable.

In this work, we fabricated an in-air superhydrophilic and underwater superoleophobic filtering membrane by the facile and inexpensive spray coating of TiO_2_ nanoparticles on a microporous stainless steel mesh substrate (TiO_2_@SSM). The advantage of this filtering membrane, besides the simplicity of its fabrication, is that TiO_2_ nanoparticles are well known photocatalysts and hence have the potential to photocatalytically purify the permeated water and also to self-clean the membrane’s surface by irradiating light. In this work, we separately studied the oil–water separation efficiency and the efficiency of the photocatalytic degradation of the coated membrane for organic pollutants. It was found that the fabricated TiO_2_@SSM membrane is water passing, showed an oil–water separation efficiency as high as 99%, and achieved a high level of stability even after ten cycles of repeated use. In the photocatalytic front, the fabricated TiO_2_@SSM membrane showed itself to be photo-responsive and achieved a 99% degradation of organic dye from water. Hence, the novelty of this work is its dual functionality with an excellent oil–water separation efficiency and photocatalytic degradation efficiency. Second, the roughness engineered surface showed a rapid transition from the extreme level of in-air oil adherence to under-water oil repellency, which is the key factor that brought about the observed oil–water separation efficiency. Additionally, the simplicity of the fabrication of the surface with an expensive material is also a novelty. With proper engineering, this membrane has the potential to be simultaneously used as a filtering medium for oil–water separation and also as a purifying agent for the permeated water, as well as the self-cleaning of the membrane.

## 2. Materials and Methods

### 2.1. Materials

Titanium dioxide nanoparticles (TiO_2_; Particle size = 10–20 nm), acetone, ethanol, methanol, hydrochloric acid, hexadecane, octane, and dodecane were commercially acquired from Sigma Aldrich (Burlington, MA, USA). A microporous stainless steel mesh substrate was acquired from TWP, Inc. USA (Burlington, MA, USA).

### 2.2. Fabrication of TiO_2_ Nanoparticles-Coated SSM Membrane

**Figure 1:**
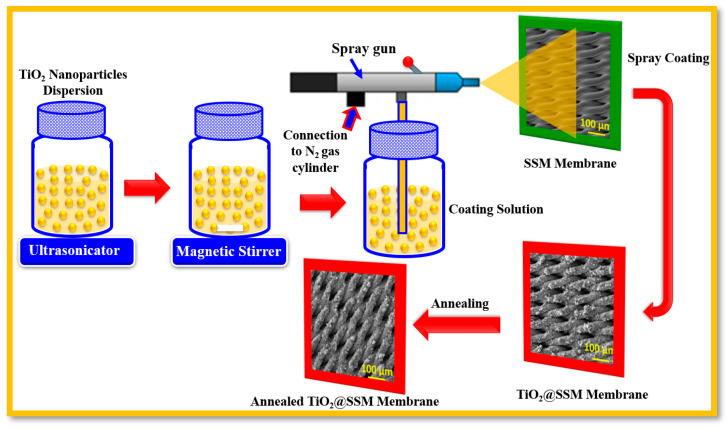
Schematic diagram for the fabrication of annealed TiO_2_@SSM membrane using spray coating approach.

The aqueous solution of commercially acquired TiO_2_ was initially subjected to ultra-sonication to remove of the lumps and also to make a homogeneous suspension. The microporous stainless steel mesh substrates (SSM) were cleaned in an ultrasonic bath with DI water and also with ethanol; they were then annealed at 500 °C for 4 h to obtain a rough surface. Subsequently, the homogeneous suspension of TiO_2_ nanoparticles was spray-coated on the clean SS mesh membrane followed by annealing at 500 °C to obtain the TiO_2_@SSM membranes. The schematic of the fabrication of TiO_2_ nanoparticles-coated SSM membranes using spray coating is shown in Figure 1. For the spray coating, a spray gun (McMaster Carr) with a nozzle diameter of 0.75 mm was used under the nitrogen pressure of 170 kPa, and 10 mL of TiO_2_ suspension was used to coat the SSM membranes. The distance between the nozzle and substrate was fixed at an optimum distance of 20 cm and the diameter of the sprayed area was 7 to 10 cm. There is a delicate balance between the spraying distance and the duration of spraying, and in our case, it was found to be 1 min to achieve the required surface roughness without blocking the pores, which doing so would diminish the permeation flux. 

### 2.3. Characterization

The XRD analysis of the uncoated SSM membrane and TiO_2_-coated SSM membranes were carried out by the benchtop Rigaku X-ray diffractometer (Rigaku, Tokyo, Japan). The surface morphology and elemental mapping analysis were conducted by field-emission scanning electron microscopy (TESCAN, Lyra3, Kohoutovice, Czech Republic), equipped with an Energy Dispersive X-ray Detector (EDX). A contact angles goniometer (KRUSS, Hamburg, Germany) was used to measure the surface wettability of the TiO_2_-coated SSM membranes.

### 2.4. Oil–Water Separation

A gravity-driven oil–water separation system was used to test the oil–water separation efficiency of the TiO_2_@SSM membranes. In total, 2 glass tubes measuring 2.5 cm in diameter and 20 cm in length were connected by keeping the TiO_2_@SSM membrane in the middle with a connecting flange, and the whole system was vertically kept using a lab stand. The top tube above the TiO_2_@SSM membrane served as the feed side where the oily water was poured, and the bottom tube, along with the 50 mL glass beaker, collected the permeate. The oil–water separation efficiency was calculated using the following formula (Equation (1)):

(1) $$Separation\;efficiency\;\left(\%\right)=1-\left[\frac{C_{p}}{C_{0}}\right]\times 100\;$$

### 2.5. Photocatalytic Dye Degradation

A broad band Xe/Hg lamp with an appropriate wavelength filter was used as the radiation source for the photocatalytic reaction; the reaction sample was 80 mL of aqueous solution of methylene blue (MB) dye. The TiO_2_@SSM membrane was immersed in the mixture and subjected to light irradiation and the irradiated sample was collected at a regular interval for quantification using the intensities of the absorbance spectrum of the dye.

## 3. Results and Discussion

### 3.1. X-ray Diffraction and Morphological Analysis

**Figure 2:**
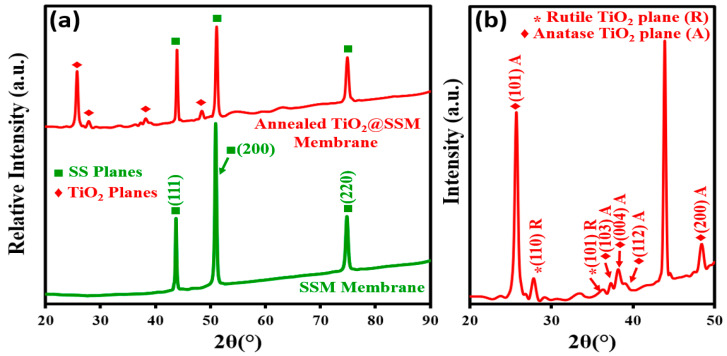
(**a**) XRD patterns of SSM membrane and annealed TiO_2_@SSM membrane. (**b**) Zoomed pattern of annealed TiO_2_@SSM membrane (**b**) from 20° to 50°.

The XRD patterns of the bare SSM and TiO_2_@SSM membrane are depicted in Figure 2a in a 2θ range between 20 and 90°, along with the enlarged XRD pattern of the TiO_2_@SSM membrane in Figure 2b. In Figure 2a, only the characteristic (111), (200), and (220) diffraction planes are present for SSM, and for the TiO_2_@SSM membrane, in addition to the above three SSM peaks, the (101), (103), (004), (112), and (200) diffraction planes pertained to the anatase phase and (110) and (101) diffraction planes due to the rutile phase of TiO_2_ being present. This indicates the successful deposition of TiO_2_ nanoparticles on the surface of microporous SSM.

**Figure 3:**
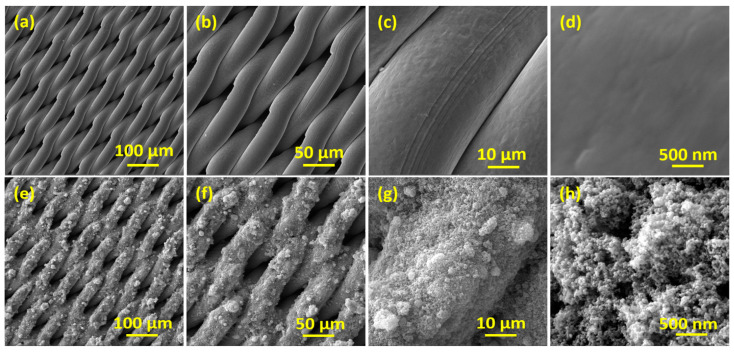
FE-SEM images of SSM membrane (**a**–**d**) annealed TiO_2_@SSM membrane (**e**–**h**) at different magnifications.

The surface morphology using FE-SEM images of the bare SSM (Figure 3a–d) and TiO_2_@SSM membrane (Figure 3e–h) representing four different magnifications are shown in Figure 3. The FE-SEM images of the bare SSM shows only the micro-porosity of the smooth SSM surface, where the images of the TiO_2_@SSM membrane show the uniform distribution of the TiO_2_ nanoparticles on the porous SSM surface. FESEM images of the TiO_2_@SSM membrane not only show the proper deposition of the TiO_2_ nanoparticles on the SSM surface but they also manifest the presence of a good surface roughness, which is essential to achieve the desired surface wettability. 

**Figure 4:**
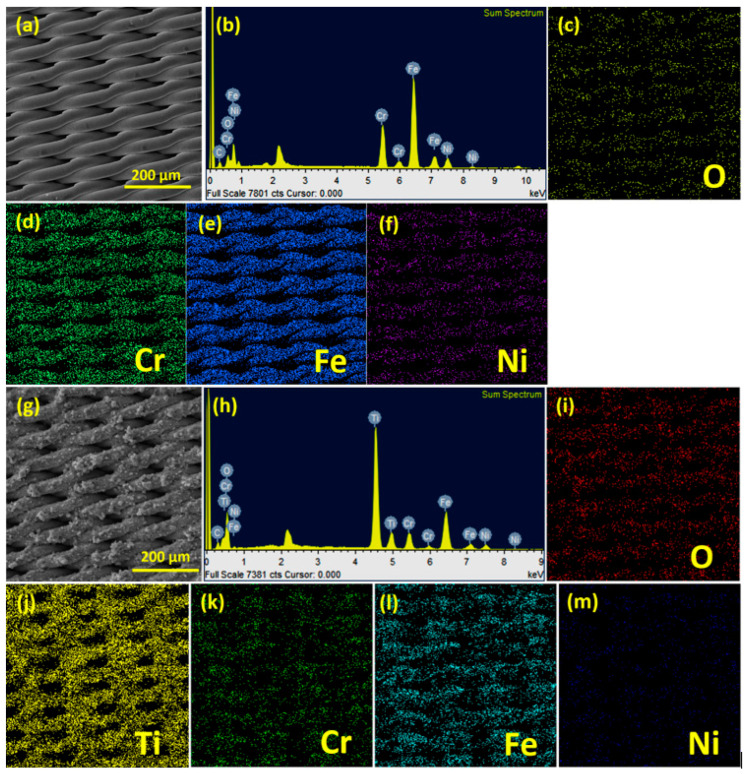
(**a**) SEM image of SSM, (**b**) EDX of SSM, Elemental mapping images of SSM for O (**c**), Cr (**d**), Fe (**e**) and Ni (**f**). (**g**) SEM image of TiO_2_@SSM membrane, (**h**) EDX of SSM, Elemental mapping images of TiO_2_@SSM membrane for O (**i**), Ti (**j**) Cr (**k**), Fe (**l**) and Ni (**m**) and elemental mapping analysis of SSM membrane and annealed TiO_2_@SSM membrane.

In addition to the morphological studies using FESEM, EDX elemental analysis and the mapping of the elements present in bare SSM (Figure 4b) and the TiO_2_@SSM membrane (Figure 4h) are presented in Figure 4. The EDX of SSM shows the presence of carbon (C), oxygen (O), iron (Fe), chromium (Cr) and nickel (Ni), the elements expected to present in the stainless steel alloy, whereas the EDX of the TiO_2_@SSM membrane shows the presence of an intense titanium (Ti) peak in addition to the reduced intensities of the elemental peaks from the SSM. The dominance of the Ti peak and the diminishing intensities of the elements present in the SSM is a clear indication of the proper coating of TiO_2_ nanoparticles on the surface of microporous SSM. The elemental distribution of the above elements in the bare SSM (Figure 4c–f) and TiO_2_@SSM (Figure 4i–m) membrane were characterized by the elemental mapping of individual elements on both of the surfaces. In the elemental mapping of the bare SSM, the distribution of iron (Fe), chromium (Cr), and nickel (Ni) were quite uniform, whereas they were weakly distributed in TiO_2_@SSM due to the heavy masking of TiO_2_ on the SSM surface. On the other hand, the distribution of Ti and O were quite dominant and uniform in TiO_2_@SSM, which further substantiates the successful coating of TiO_2_ nanoparticles on the SSM surface. 

### 3.2. Surface Wettability of TiO_2_ Nanoparticles-Coated SSM

**Figure 5:**
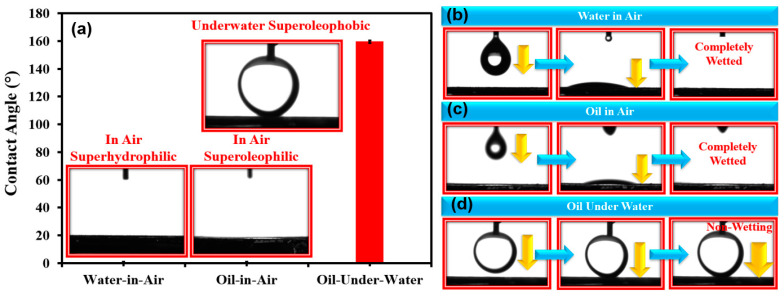
(**a**) Surface wettability analysis of annealed TiO_2_@SSM membrane for water (in-air) and oil (in-air and under water). (**b**) Different steps of water contact angle analysis of annealed TiO_2_@SSM membrane in-air showing water is approaching, touching, and completely wetted to the surface, (**c**) different steps of oil contact angle analysis of annealed TiO_2_@SSM membrane in-air showing oil is approaching, touching, and completely wetted to the surface, (**d**) different steps of oil contact angle analysis of annealed TiO_2_@SSM membrane under water showing oil is approaching, beading, and repelling (non-wetting) to the surface.

The wettability of the oil and water on the membrane’s surface in an air and water environment is an important factor that decides the functionality of the membrane as a medium for oil–water separation. The relative dominance of the surface energy of the membrane’s surface and the surface tension of the liquid medium, along with the dimension and the distribution of surface roughness, are quite instrumental in deciding the wettability of the surface for the oil and water on the surface. The wettability is experimentally quantified using the contact angles that the particular liquid makes on the solid surface in an air or water environment. Figure 5a shows the photographic images of the water and oil droplets on the annealed TiO_2_@SSM membrane in air, water, and oil and in a water medium for oil. It is quite clear that in an air environment, both oil and water droplets completely spread on the surface, yielding both water-in-air (θ_WA_) and oil-in-air (θ_OA_) contact angles close to zero, which indicates that the surface is superoleophilic and superhydrophilic in air. In air, the surface energy of the TiO_2_@SSM membrane is more than the surface tension of oil and water, making the more adhesive force enabling for both oil and water to spread on the membrane’s surface. In the case of the oil wettability of the surface under water, from Figure 5a, it is quite clear that the oil beads on the membrane’s surface rather than spreading out, measuring the oil-in-water contact angle (θ_OW_) to be as high as ~160°. This indicates that the surface switches from being superoleophobic in air to being superoleophobic in water. Under water, due to the superhydrophilicity of the membrane, a layer of water film on the membrane’s surface weakens the surface energy of the membrane relative to the surface tension of oil; the cohesive force of the oil is more than the adhesive force of the surface to make the oil bead on the surface. Additionally, Figure 5b–d show the temporal evolution of oil and droplets on the surface in air and under water, where it is quite clear in an air environment that both oil and water droplets spread in no time and the oil droplet in the water environment remains beading for a long time. In addition to the surface energies, the surface roughness also plays an important role in modifying the wettability of the surfaces according to the original Young’s law of wettability, modified by Wenzel and Cassie Baxter [14,15]. These wettability conditions amount to the fact that when oily water comes in contact with the TiO_2_@SSM membrane, the water phase is attracted to the membrane due to superhydrophilicity and the oil phase in water is vehemently repelled by the membrane due to the superoleophobicity of the membrane under water. The thickness of the resultant TiO_2_@SSM membrane was also measured and was found to be ~475 µm. 

### 3.3. Oil–Water Separation Performance

**Figure 6:**
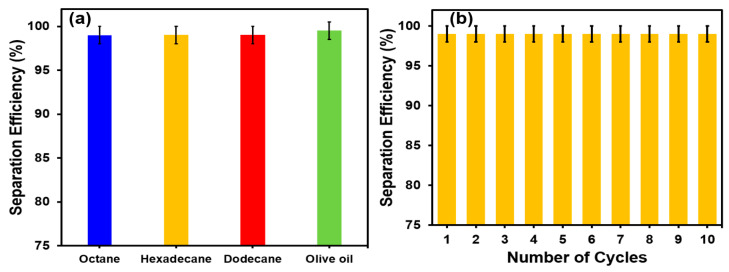
(**a**) Oil–water separation performance of annealed TiO_2_@SSM membrane for different oil–water mixtures and (**b**) stability of annealed TiO_2_@SSM membrane in terms of separation efficiency during cyclic experiment.

The TiO_2_@SSM membrane with the unique oil and water wettability showed an excellent performance for oil–water separation in a gravity-driven separation system. The gravity-driven oil–water system consists of two glass tubes of a one-inch diameter, connected through the circular TiO_2_@SSM membrane by joining clamps and fixed in a vertical stand. The upper tube, above the membrane, is the feed side, where the oil–water mixture is poured and the bottom tube below the membrane is the permeate side through which the water permeates and falls in a beaker. When the oily water is poured into the feed side, the bulk of the water tends to stay momentarily above water, and the small oil droplets in the water then come into contact with the water-drenched membrane; the oil droplets are strongly repelled by the surface due to its under-water superoleophobicity, and at the same time, the pure water easily becomes permeated through the membrane due to superhydrophilicity. The oil and water are mixed in an equal ratio and after passing through the TiO_2_@SSM membrane, the permeate is expected to have only a water phase as most of the oil droplets are rejected by the membrane’s surface in the feed side. However, a small quantity of oil still remains in the water, and the amount of the residual oil is estimated by thermogravimetry to calculate the oil–water separation efficiency. The bar chart in Figure 6a shows the oil–water separation efficiency of octane, hexadecane, dodecane, and olive oil mixed in water, and for all the oil–water mixture under study, the separation efficiencies are close to 99%. In addition to the separation efficiency, the stability and the robustness of the TiO_2_@SSM membrane was verified by estimating the oil–water separation efficiency in every consecutive trial of oil–water separation by reusing the same membrane.

**Figure 7:**
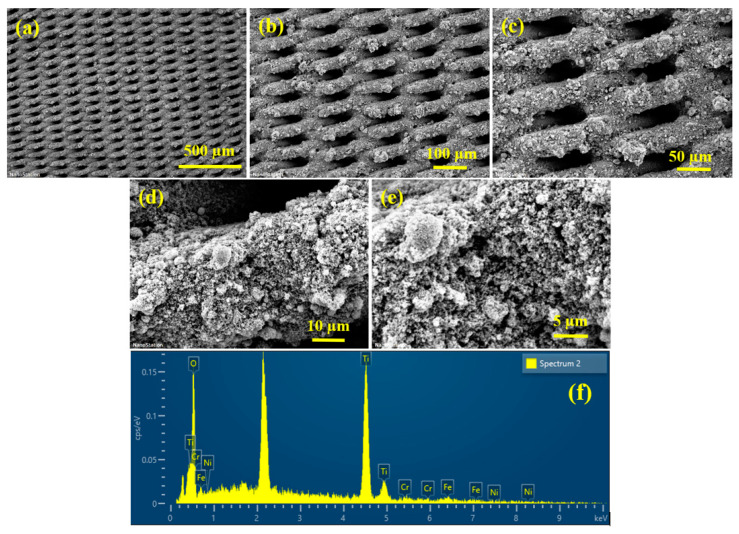
(**a**–**e**) SEM images of annealed TiO_2_@SSM membrane and (**f**) EDX of annealed TiO_2_@SSM membrane.

It is quite clear from Figure 6b that the same level of 99% oil–water separation efficiency is retained in every trial of 10 consecutive cycles. This level of stability of the TiO_2_@SSM membrane is possible only if the membrane strongly adheres to the stainless steel surface, maintaining the same level of surface roughness and retaining its structural stability. In order to evaluate the mechanical stability of the membrane, the SEM and EDX images of the membrane after repeated use were taken and they are shown in Figure 7. It is quite obvious that in the used membrane, the adhesion of the TiO_2_ nanoparticles on the SSM surface is as good as the new membrane (Figure 7a–e). It is also obvious from EDX (Figure 7f) that the dominance of the Ti peak and the diminishing intensities of the elements present in the SSM is a clear indication of the exitance of the TiO_2_ nanoparticles coating the surface of the microporous SSM. Hence, the membrane is mechanically very robust. The excessive oil–water separation efficiency of the TiO_2_@SSM membrane stems from the unique wettability accomplished on the membrane’s surface due to the characteristics of the TiO_2_ nanoparticles, and also the surface roughness achieved by spray coating. 

**Figure 8:**
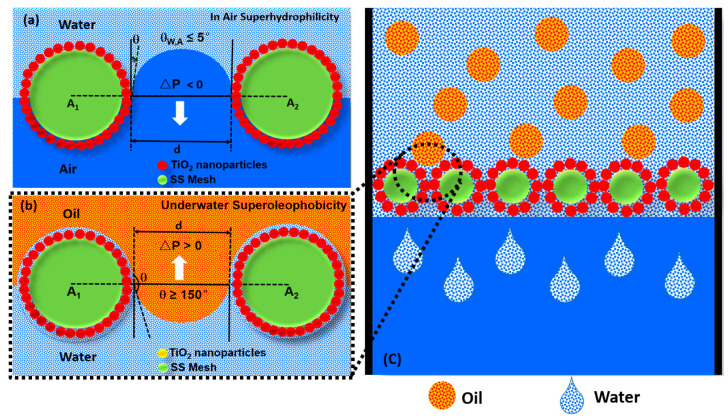
(**a**) in air superhydrophilicity, (**b**) underwater superoleophobicity and (**c**) use of in air super-hydrophilic and underwater superoleophobicity TiO_2_@SSM membrane for oil-water separation.

The under-water superoleophobicity with a high oil-in-water contact angle (θ_OW_) can be achieved by the optimum interfacial surface energies of the surface–water–air interface (γ_WA_), surface–oil–air interface (γ_OA_), and surface–oil–air interface (γ_OW_). According to the Young–Dupre equation of wettability for oil–surface–water interfaces (Equation (1)), γ_WA_ should be greater than γ_OA_ and at the same time, γ_OW_ should be low enough to achieve a high level of under-water oil repellency. As shown in Figure 8, the superhydrophilic (θ_WA_ = 0) and under-water superoleophobic (θ_OW_ = 160°) TiO_2_@SSM membrane with the pore diameter leads up to a positive upward oil pressure (△P2) due to solid–water–oil interfacial tension (Figure 8a) and a negative downward water pressure (△P1) due to solid–water–air interfacial tension (Figure 8b) in accordance with the Young–Laplace equation (Equations (2) and (3)) [14,15].

△P1 = (−2γ_WA_·cosθ_WA_)/d (2)

△P2 = (−2γ_OW_·cosθ_OW_)/d (3)

The positive upward pressure of the oil and the negative downward pressure of the water pave the way for the water to pass and the oil to be rejected by the membrane. The oil in the feed side exerts a downward hydrostatic pressure, which is overpowered by the upward oil pressure (△P2) and as the height of oil column increases, the hydrostatic oil pressure becomes greater than △P2; under this condition, the oil retaining efficiency of the membrane fails and oil starts flowing through along with water. So, in order make this retention pressure high, the membrane surface should be so engineered to yield a high oil repellency by selecting the material with the optimum interfacial surface energies, surface roughness, and pore size.

### 3.4. Photocatalytic Performance of TiO_2_@SSM Membrane

**Figure 9:**
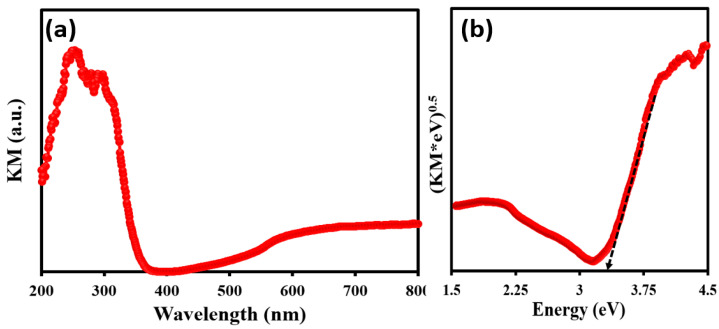
(**a**) UV-DRS of annealed TiO_2_@SSM membrane. (**b**) Tauc plot of annealed TiO_2_@SSM membrane.

The second functionality of the fabricated membrane is its potential to function as a photocatalyst under light irradiation to self-clean the membrane surface and also disinfect the organic pollutants present in the permeated water from the oil–water separation. The light responsive property of the coated TiO_2_ is presented in Figure 9a, where the maximum absorption is in the spectral range between 200 nm and 400 nm. The spectra in Figure 9a is obtained from the diffuse reflectance spectra of the coated membrane, from where it is transformed into the absorption equivalent Kubelka Munk function (KM), using the relation KM = (1 − R)^2^/2R, where R is the reflectance. In addition to this, the band gap energy of the material is also estimated using the Tauc plot, which is basically the (KM × hʋ)^0.5 vs.^ hʋ plot, whose x-intercept directly presents the band gap energy. This is validated from the well-known relation (αE)^(1⁄n)^ = A(E − Eg) in the field of the semiconductor where A is a frequency independent constant, n takes the value of 2 in the above equation, and a value of 0.5 is provided in the Tauc plot for an indirect band gap material. TiO_2_ is an indirect band gap material, where the valance and conduction bands are in a different momentum space and hence require the intervention of a phonon to conserve momentum and make the electronic transition possible. The Tauc plot for the coated membrane is presented in Figure 9b and the estimated band gap is around 3. 3 eV. The optical studies indicate that the coating is photo-responsive. 

**Figure 10:**
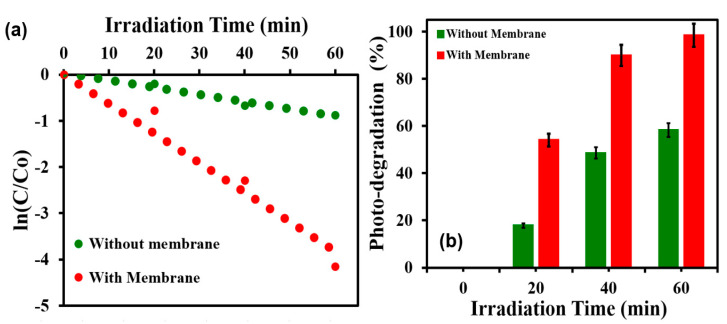
(**a**) Decay curve of photocatalytic degradation of organic dye (MB dye) with and without annealed TiO_2_@SSM membrane. (**b**) Photocatalytic degradation % of MB dye with and without annealed TiO_2_@SSM membrane with time.

The photocatalytic degradation is exponentially decayed with time; hence, the decay curve was linearized and the extent of the deactivation of the methylene blue dye with the exposure time is conveniently quantified by the slope of the ln N/N_0_ versus the exposure time. The photocatalytic degradation of methylene blue dye with the TiO_2_@SSM membrane is shown in Figure 10a, where the two decay curves are represented in the presence and absence of TiO_2_@SSM. It is quite clear from Figure 10a that the photocatalytic degradation with the TiO_2_@SSM membrane under light irradiation shows a curve with a much higher slope than the one without the membrane, which indicates that the membrane is capable of photocatalytically degrading the organic pollutants in addition to its capability for oil–water separation. The marginal slope (dye degradation) observed in the curve without the TiO_2_@SSM membrane is simply the light-induced (non-photocatalytic) degradation. Figure 10b shows the percentage of photocatalytic degradation of methylene blue dye using the TiO_2_@SSM membrane at different irradiation times. 

**Figure 11:**
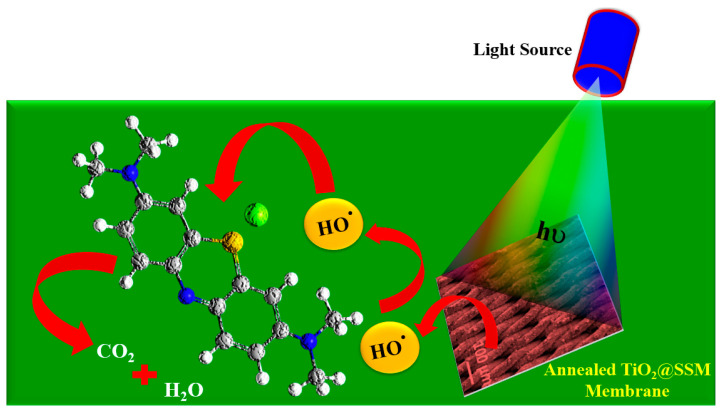
Schematic diagram of photocatalytic degradation of hazardous dye using annealed TiO_2_@SSM membrane under UV light irradiation.

The schematic of the photocatalytic deactivation of the TiO_2_@SSM nanocomposite is depicted in Figure 11. When the light with a wavelength shorter than the wavelength represents the band gap of the coated material, the electron hole pairs (e^−^/h^+^) are generated on the TiO_2_ surface as the electron transfers from the conduction band to the valance band. The holes in the valance band of TiO_2_ are more positive than the reduction potential of water, hence the positively charged holes oxidize the water molecule and produce hydroxyl radical and hydrogen ion and two hydroxyl radicals (OH^•^) combine to form an oxygen molecule. The oxygen molecule is reduced by the electrons in the conduction band of TiO_2_ to form a super oxide radical (-O2^−^). The highly reactive hydroxyl radical and the superoxide generated by the redox reaction mediated by photo-induced charge carriers degrade the organic pollutants.

In order to highlight the superior performance of the TiO_2_@SSM membrane in terms of the oil–water separation efficiency and photocatalytic degradation efficiency, a comparison of these two parameters is made with other similar works in Table 1. Despite the fact that in some of the cited references [19,20,21,22], the oil–water separation efficiencies are comparable to the performance of the TiO_2_@SSM membrane, the photocatalytic degradation efficiencies in some of the works went down to 85%. Therefore, from Table 1, it is quite evident that the TiO_2_@SSM membrane shows a better oil–water separation efficiency than the membranes listed and it exhibits the best photocatalytic degradation efficiency. As the TiO_2_@SSM membrane possesses an excellent oil–water separation efficiency and photocatalytic degradation, this is ideal for the simultaneous separation and purification of oily water. 

## 4. Conclusions

A water passing, light-responsive, oil–water separation membrane (TiO_2_@SSM) possessing in-air superhydrophilicity, in-air superoleophobicity, and under-water superoleophobicity was fabricated by spray coating TiO_2_ nanoparticles on a stainless steel membrane, followed by annealing at 500 °C. Structural and morphological characterization using XRD, FE-SEM, and EDX revealed that the TiO_2_ nanoparticles were well placed on the stainless steel membrane. The contact angle measurements using goniometric showed that the fabricated TiO2@SSM membrane surface showed that the contact angles on the surface–water–air interface and surface–oil–air interface are 0° (superhydrophilic and superoleophilic), and the surface–oil–water interface is close to 160° (superoleophobic under the water). The TiO_2_@SSM membrane was used as a separation medium in the gravity-driven oil–water separation system and an oil–water separation efficiency as high as 99% was achieved. Additionally, TiO_2_@SSM was used as a photocatalyst for the degradation of methylene blue dye present in water, and it was found to be close to 100% degradation of dye. This would usher in an unprecedented dual-purpose system that not only removes oil but also clears up the harmful microorganism in it as well, marking an exciting technological revolution in the oil–water separation industry.

## Figures and Tables

**Table 1:** Comparison of oil–water separation efficiency and photocatalytic performance (pollutant degradation efficiency) with other reported literatures.

Material Used	Wetting Behavior	Contact Angle	Oil–Water Separation Efficiency (%)	Photocatalytic Activity (%)	Refs.
Ag_2_O/TiO_2_@CuC_2_O_4_ nanocomposite-coated mesh	Superhydrophilic and underwater superoleophobic	WCA (in-air) = ~0° OCA (under water) = ~150°	~95%	~94% degradation of MB dye in 60 min	[19]
BiVO_4_-coated mesh	Superhydrophilic and underwater superoleophobic	WCA (in-air) = ~0° OCA (under water) = ~159°	~98.6%	~85% degradation of MB dye in 200 min	[20]
Zn-Ni-Co LDHs@NiMoO_4_-coated mesh	Superhydrophilic and underwater superoleophobic	WCA (in-air) = ~0° OCA (under water) = ~164.9°	~99%	~93.95% degradation of MB dye in 80 min	[21]
W, N-co-doped-TiO_2_ nanobelts (WNTNBs)-coated mesh	Superhydrophilic and underwater superoleophobic	WCA (in-air) = ~0° OCA (under water) = ~150°	~99.5%	~94.3% degradation of MB dye in 180 min	[22]
TiO_2_@SSM membrane	Superhydrophilic and underwater superoleophobic	WCA (in-air) = ~0° OCA (under water) = ≥160°	99%	98.43% degradation of MB dye in 60 min	This Work

## Data Availability

Data is contained within the article.
